# Recombinant Bispecific Antibodies to the Human ErbB2 Receptor and Interferon-Beta

**DOI:** 10.32607/actanaturae.10903

**Published:** 2020

**Authors:** A. A. Panina, V. S. Rybchenko, O. N. Solopova, D. S. Balabashin, S. A. Yakimov, T. K. Aliev, D. A. Dolgikh, P. G. Sveshnikov, M. P. Kirpichnikov

**Affiliations:** Shemyakin-Ovchinnikov Institute of Bioorganic Chemistry, Russian Academy of Sciences, Moscow, 117997 Russia; Lomonosov Moscow State University, chemical faculty, Moscow, 119991 Russia; Russian Research Center for Molecular Diagnostics and Therapy, Moscow, 117149 Russia; Federal State Budgetary Institution «N.N. Blokhin National Medical Research Center of Oncology» of the Ministry of Health of the Russian Federation, Moscow,115478 Russia; Lomonosov Moscow State University, biological faculty, Moscow, 119991 Russia

**Keywords:** bispecific antibodies, CrossMab, ErbB2, interferon-beta, immunocytokine complex

## Abstract

The development of and research into new therapies that can selectively and effectively destroy tumor cells that overexpress the ErbB2 receptor is a pressing task. Recently, research into the use of type I interferons in the treatment of cancer has intensified. Cytokine therapy is aimed at activating the cells of the immune system to fight tumors, but it has drawbacks that limit its use because of a number of side effects the severity of which varies depending on the dosage and type of used cytokine. At the moment, a number of studies are being conducted regarding the use of IFNβ in oncology. The studies are aimed at mitigating the systemic action of this cytokine. The immunocytokine complex made of a bispecific antibody against the ErbB2 receptor and recombinant IFNβ developed in this study underlies the mechanism meant to avoid the systemic action of this cytokine. Part of this study focuses on the development of full-length antibodies that bind to the ErbB2 receptor on the one hand, and bind and neutralize IFNβ, on the other hand, which allows us to consider the antibodies as a means of cytokine delivery to tumor cells.

## INTRODUCTION

Breast cancer is the leading cause of cancer mortality in females. It accounts for almost 11% of all cancers and is the most prevalent cancer in the world. In the Russian female population in 2017, breast cancer accounted for 21.1% of malignant neoplasms; the number of patients with stage I–II of the disease amounted to 69.9% [1]. Overexpression of the epidermal growth factor receptor ErbB2 was detected in a significant percentage of the tumors. Amplification and/or overexpression of ErbB2 occurs in 20–34% of invasive breast cancers [2, 3]; it is associated with increased cell proliferation, enhanced angiogenesis, decreased apoptosis of tumor cells, and, as a result, with a high metastasis potential [4, 5]. Overexpression of ErbB2 is considered an independent prognostic factor that denotes an increased risk of disease recurrence. In the case of stage I–II ErbB2-positive breast cancer, the risk of local recurrence and the risk of distant metastasis are 2.7-fold and 5.3-fold higher, respectively, than that of a ErbB2-negative cancer [2]. In addition, ErbB2 can be overexpressed in tumors of the bladder, pancreas, ovary, uterus, colon, kidney, head and neck, stomach, esophagus, and prostate [6]. Overexpression of HER2 is detected mainly in malignant neoplasms of epithelial origin [7]. The HER2/neu gene status (ErbB2) is one of the main indicators used to identify breast cancer subtypes, predict disease progression, and choose treatment options for patients. Thus, the relationship between overexpression and/or amplification of ErbB2 and a poor clinical prognosis suggests that ErbB2 is an important link in the molecular biological classification of breast cancers and an important therapeutic target. Currently, there are drugs whose action targets ErbB2. A breakthrough in antitumor therapy occurred with the advent of the drug Herceptin (trastuzumab), which is a humanized antibody to the extracellular domain of ErbB2 [8-10], which inhibits the proliferation of tumor cells. The efficacy of trastuzumab monotherapy is 26–35% in previously treated patients with metastatic ErbB2-positive breast cancer and 12–15% in patients who have not undergone previous therapy for metastasis [11]. Currently, trastuzumab, in combination with chemotherapy, is considered the main drug for ErbB2-positive breast cancer [12]. However, there is resistance to this drug in some cases. Therefore, the search for new therapies for ErbB2-positive tumors remains an important area of research.

Human interferon-β (IFNβ) is an immunomodulatory cytokine exhibiting antiviral, antiproliferative, pro-apoptotic, and anti-angiogenic activity. The efficacy of the antiproliferative and apoptotic action of interferons varies depending on the type of tumor cells; however, IFNβ is considered more effective than IFNα and IFNγ, e.g., in the inhibition of hepatocellular carcinoma [13], glioma [14], and pancreatic [15] and breast [12, 16] tumors. IFNβ facilitates the arrest of tumor cells in S-G2-M cell cycle phases and also stimulates apoptosis in them [15]. In addition, experimental studies have shown that IFNβ induces the expression of major histocompatibility complex class I (MHC-I) molecules, which is considered one of the universal mechanisms for enhancing the antitumor response due to T-cell cytotoxicity [17]. The mechanism of action of type I interferons (IFNα and IFNβ), as well as current ideas about the use of interferons in cancer therapy, is addressed in detail in review [18].

A potentially promising and attractive cytokine therapy is rooted in activating the cells of the immune system to fight tumors, but its use is limited by its side effects, the severity of which varies depending on the dose and type of the cytokine. The possibility of reducing the systemic effect of IFNβ in the treatment of tumors is currently under study. Most of these studies use viral vectors carrying the IFNβ gene. A number of studies have shown that type I interferons conjugated with monoclonal antibodies to tumor cell-associated proteins can induce an antiproliferative effect caused by both the conjugated interferon and the antibodies [19-23]. Bi- and multi-specific antibody derivatives may be considered as the next, and very promising, generation of biologics for targeted cancer therapy. The general concept behind these antibodies is combination of two recombinant antibodies with different specificities to interact with at least two antigens or epitopes.

We plan to create an immunocytokine complex where one of the bispecific antibody parts binds to IFNβ and neutralizes its effect, and the second should be able to bind to the ErbB2 receptor on the tumor cell surface. IFNβ is supposed to locally accumulate at the tumor and metastasis sites, which excludes the adverse systemic reactions underlying the clinical picture of the side effects typical of IFNβ monotherapy.

The aim of this study was to create, based on the previously generated recombinant chimeric neutralizing mAbs to IFNβ [24] and an antibody to ErbB2, bispecific full-length antibodies as a component of the immunocytokine complex with IFNβ, which is intended for the treatment of ErbB2-positive tumors, as well as to study their biochemical and immunochemical properties.

## EXPERIMENTAL

We used the SKOV3-ErbB2 human ovarian adenocarcinoma cell line expressing ErbB2 (ATCCR HTB-77™); the HT29 human colon adenocarcinoma cell line with a low or without expression of ErbB2c (ATCCR HTB-38™); a SKOV3 human ovarian adenocarcinoma cell line that had lost its overexpression of ErbB2.

We used a pharmaceutical substance of glycosylated IFNβ-1a manufactured by LLC Pharmapark, the commercial drug IFNβ-1b (Betaferon), a nitrocellulose membrane (nitrocellulose membrane filters with a pore size of 0.45 μm, S045A330R, Advantec MFS, Inc., USA); a Vivaflow 200 membrane (Sartorius Stedim Biotech, Germany), 96-well high-binding capacity plates (Corning- Costar, Netherlands), and Twin 20.

Preparation of nucleotide sequences encoding the L and H chains of anti-human ErbB2b antibodies with knob* and hole mutations

Nucleotide sequences encoding the VL and VH antibodies to ErbB2 were synthesized by a chemical-enzymatic procedure using overlapping oligonucleotides based on the amino acid sequences of the antibody trastuzumab [25], with allowance for an optimization of the codon frequency in the Chinese hamster genome. The Kozak sequence and leader peptide sequences were placed at the 5’-ends of the antibody VL and VH sequences to secrete the antibodies into the culture fluid. Also, for subsequent matching of the VL and kappa domains, as well as the VH and constant CH1–H3 domain sequences, segments complementary to the 5’-fragment of the constant domains were introduced at the 3’-end of the V domain sequences. The VH and VL sequences were combined using SOE-PCR. Knobtype* and hole-type mutations were introduced into the CH_3_ domain of the H chain, using mutagenizing primers.

**Preparation of sequences encoding the L and H chains of the IFNβ-neutralizing B16 antibody with a crossover**

To prepare antibody genes with a crossover, additional sequences were introduced into the antibody VL and VH sequences using PCR. The primers were designed based on the VL and VH sequences. To prepare the L chain gene with a crossover, the elbow region and a small fragment of the H chain sequence containing the Bsp120I restriction endonuclease recognition site were introduced at the 5’-end of the VL chain sequence. Then, a fragment overlapping with the kappa L chain constant domain sequence was introduced into the B16 antibody VH chain sequence by PCR. The kappa domain sequence was fused with the modified VH domains using SOE-PCR.

To generate the H chain gene with a crossover, a sequence containing an H chain CH1 domain fragment and the Bsp120I restriction site was introduced at the 3’-end of the VL chain sequence. Then, the produced sequences were cloned into an expression vector at the NheI and Bsp120I restriction sites, which was prepared by restriction at the NheI and Bsp120I sites of a plasmid containing the anti-ErbB2 antibody H chain gene with a knob-type* mutation.

**Construction of bicistronic expression plasmids containing B16 antibody genes with crossover and KiH-star mutations**

Bicistronic expression vectors were prepared by ligation of a sequence of the IRES element and H chain gene into plasmids containing the L chain gene. This vector was generated for each component of a bispecific antibody. The IRES element sequence flanked by BamHI and NheI restriction sites, after treatment with the restriction enzymes BamHI and NheI and isolation of a 630 bp fragment from a 1% agarose gel, was ligated with a vector site containing the antibody L-chain gene with crossover (BamHI–XhoI) and a fragment involving the H-chain gene with crossover and knob-type* mutations.

**Preparation of experimental bispecific antibody samples**

Transient expression was used to produce test antibody samples. Twenty-four hours before transfection, CHO cells at a concentration of 4 × 10^6^ cells/mL were transferred to an Erlenmeyer flask containing 30 mL of a CD OptiCHO culture medium (Invitrogen, USA) supplemented with 8 mM *L*-glutamine (Invitrogen) and 0.1% Pluronic F68 (Gibco, USA). Cell transfection was performed using lipofectamine (Invitrogen) according to the manufacturer’s recommendations and pairs of bicistronic vectors containing the L and H chains of the Tz antibody and B16 antibody. For transfection, we used 18 μg DNA and 15 μL of the FreeStyle MAX transfection reagent (Invitrogen). Cultivation was continued for 10–4 days at a temperature of 37°C in an atmosphere containing 8% CO_2_, under constant stirring on an ELMI S3.20L orbital shaker at 130 rpm until the amount of living cells in the culture had decreased to a level of 0.3 × 10^6^ cells/mL. To produce the required amounts of the protein, transfection was performed in six flasks for each of the plasmid pairs. After cultivation, the cells were precipitated by centrifugation at 1 200 rpm for 10 min; then, the supernatant containing antibodies was centrifuged at 4 000 rpm for 15 min, and the antibody solution was sterilized by filtration through a membrane with a pore size of 0.22 μm. Before isolation of the antibodies, the supernatant was stored at 4°C.

**Isolation of BsAbs by affinity chromatography**

Culture fluid samples were dialyzed against PBS and concentrated on a 5 mL chromatographic column packed with the MabSelect SuRe LX Protein A resin (GE Healthcare) for further purification. Antibodies were isolated according to the manufacturer’s protocol. After purification on an affinity column and, when necessary, concentration of the sample, analytical gel filtration chromatography was performed on the Superdex 200-10/300 GL resin to determine the presence and ratio of monomeric and oligomeric antibody forms. The concentration of target proteins was determined spectrophotometrically using a NanoPhotometer P300 (IMPLEN, Germany).

**Indirect enzyme immunoassay (ELISA)**

The ability of the produced proteins to interact with IFNβ or the recombinant extracellular domain of ErbB2 was determined using indirect ELISA. Antigens in PBS buffer (0.5 μg/mL) were adsorbed onto a plate, blocked with 5% bovine serum albumin (BSA) in PBS, and washed. Then, the test proteins were added, incubated at room temperature for 1 h, and washed with a 0.05% Tween 20 solution in PBS. After washing, the 4G7 monoclonal antibody to the kappa light chain domain of human Ig (LLC Bialexa, Russia), conjugated with horseradish peroxidase, was added at a dilution of 1 : 75 000, incubated at room temperature for 1 h, washed with a 0.05% Tween 20 solution in PBS, and added to a chromogenic substrate (tetramethylbenzidine). After color development, the reaction was stopped by adding 10% sulfuric acid. Optical absorption was measured at 450 nm.

**Immunoblotting of glycosylated and non-glycosylated human interferon-β using bispecific antibodies**

After electrophoretic separation of IFNβ samples in 15% PAG under non-reducing conditions, the proteins were electro-blotted onto a nitrocellulose membrane (pore size 0.45 μm; Advantec MFS, Inc., USA). Transferred proteins were detected using indirect ELISA (immunoblotting) after blocking with 5% casein at room temperature on a shaker for 1 h and washing with PBS-T (10 mM K_2_HPO_4_, pH 7.5; 0.145 M NaCl; 0.05% Tween 20). The membrane was cut into strips, placed in antibody solutions (5 μg/mL), and incubated on a shaker at room temperature for 1 h. After triple washing, the strips were incubated with horseradish peroxidase conjugated with 4G7 mAb against a human IgG kappa chain at room temperature on a shaker for 1 h. After repeated washing three times in PBS-T and final washing once in PBS, a substrate (3,3-diaminobenzidine, 4-chloro-1-naphthol, and hydrogen peroxide) was added, incubated for 10 min, and the reaction was stopped by washing the strips with water.

**Indirect ELISA using cell lysates**

To prepare lysates, culture flasks or Petri dishes were placed on ice, washed once with cold PBS, and cells were detached with a cell-culture scraper. The cell pellet was washed twice with cold PBS by centrifuging at 2 000 rpm and 4°C for 10 min. The cell pellet was lysed with a RIPA buffer supplemented with protease inhibitors (1-mM PMSF and 1-mM aprotinin). The RIPA buffer composition was as follows: 20 mM Tris- HCl (pH 7.5), 150 mM NaCl, 1 mM Na2EDTA, 1 mM EGTA, 1% NP-40, 1% sodium deoxycholate, 2.5 mM sodium pyrophosphate, 1 mM β-glycerophosphate, 1 mM Na3VO4, and 1 μg/mL leupeptin. The pellet was sonicated to maximally extract membrane proteins and then centrifuged at 12 000 rpm and a temperature of 4°C for 10 min. Supernatants were collected, and the protein concentration was measured in them; supernatant aliquots were stored at –0°C. For ELISA, the above-described procedure was used with some variations. Cell lysates were adsorbed onto a plate at a concentration of 10 μg/mL in PBS (0.01 M KH_2_PO_4_, 0.1 M NaCl), pH 7.2–7.4, 50 μL per well in a 96-well ELISA plate at 4°C overnight. The plate was washed thrice with PBS-T (0.1% Tween 20), 200 μL per well. BsAb samples were titrated from 800 ng/mL in a dilution of 2 in PBS–T (0.01 M KH_2_PO_4_, 0.1 M NaCl, 0.2% BSA, 0.1% Tween 20). The plate was incubated at room temperature for 1 h. Washing, the reaction with the conjugate, development, and measurement of optical absorption were performed as described above. The antibody trastuzumab (Herceptin) at a concentration of 10 μg/mL was used as a positive control.

**Detection of simultaneous binding of interferon-β and ErbB2 by sandwich ELISA**

Bispecific binding of the produced antibody was confirmed using sandwich ELISA in two variants. In the first variant, IFNβ (1 μg/mL) was adsorbed onto a solid phase in an amount of 100 μL/well and, after blocking, incubated with BsAbs (1 μg/mL) in 3-fold serial dilutions. After washing of the unbound antibodies, a biotinylated recombinant ErbB2 (200 ng/mL) and an avidin-horseradish peroxidase conjugate (150 ng/mL) were added. In the second variant, a recombinant extracellular domain of ErbB2 (200 ng/mL), BsAbs (also in serial dilutions), biotinylated IFNβ (250 ng/mL), and an avidin-horseradish peroxidase conjugate (150 ng/mL) were sequentially adsorbed onto the solid phase. As a control, we used the full-length B16 antibody to IFNβ and monospecific antibodies to ErbB2: the commercial drug Herceptin and the produced recombinant antibody trastuzumab (Herceptin analogue).

**Determination of the neutralizing activity of BsAbs**

The human HT29 colon adenocarcinoma cell line not expressing ErbB2 was used for the analysis. Human peripheral blood mononuclear cells (PBMCs) were isolated from the whole blood of a healthy donor in a ficoll-1077 gradient according to the procedure in [26]. Serial dilutions of BsAbs and control antibodies (mouse B16 antibody, chimeric and humanized B16-based IFNβ-neutralizing antibodies (positive control)) were prepared. Recombinant glycosylated IFNβ (Pharmapark) at a concentration of 3 ng/mL was added to the antibodies. All solutions were prepared in a complete growth medium containing 2% cattle serum. Tumor cells were cultured in a mixture with PBMCs in the presence of IFNβ and antibodies at various concentrations for 5 days. The final concentrations of active substances and cells were as follows: antibodies – from 0 to 100 μg/mL; IFNβ – 1 ng/mL (0 in control wells); PBMCs – 50 × 10^3^ cells/well; tumor – 3,000 cells/well. The number of living cells was evaluated by the MTT test [27]. Each measurement was performed in quadruplicates.

## RESULTS AND DISCUSSION

**Design of bispecific antibodies and their expression**

Co-expression of two different immunoglobulin-heavy chain genes (H_1_ and H_2_) can result in three types of heavy-chain dimers: H1–H1, H2–H2, and H_1_–H_2_. In this case, H_1_–H_1_ and H2–H_2_ are part of monospecific antibodies, and H_1_–H_2_ is part of bispecific antibodies. Given the same dimer formation efficiency, the maximum possible BsAb yield is 50% of total produced immunoglobulins. To increase BsAb production, mutations that increase the free energy of monospecific antibody formation can be introduced. This approach renders the BsAb formation energetically more favorable, which may increase the production of BsAbs compared to that of monospecific antibodies. One of the possible approaches is to generate knob-into-hole mutations [28]. In this approach, the T366W mutation is introduced into the H chain CH_3_ domain of one antibody, and the T366S, L3638A, and Y407V mutations are introduced into the H chain CH_3_ domain of another antibody. The T366W mutation leads to the emergence of a large hydrophobic base on the interface of an H chain CH_3_ domain dimer. This mutation will hereinafter be referred to as the knob-type. T366S, L3638A, and Y407V mutations lead to the formation of a sterically complementary hole for T366W. To facilitate the purification of BsAbs by affinity chromatography, the H457R and Y458F mutations were introduced into the H chain CH_3_ domain containing knob-type mutations. These mutations change the formation constant of the antibody– staphylococcus A protein complex, which enables the separation of a mixture of bispecific and monospecific antibodies during elution with a pH gradient buffer from an affinity carrier [29]. Further in the text, the H457R and Y458F mutations will be designated as “star” or “*”.

**Fig. 1:**
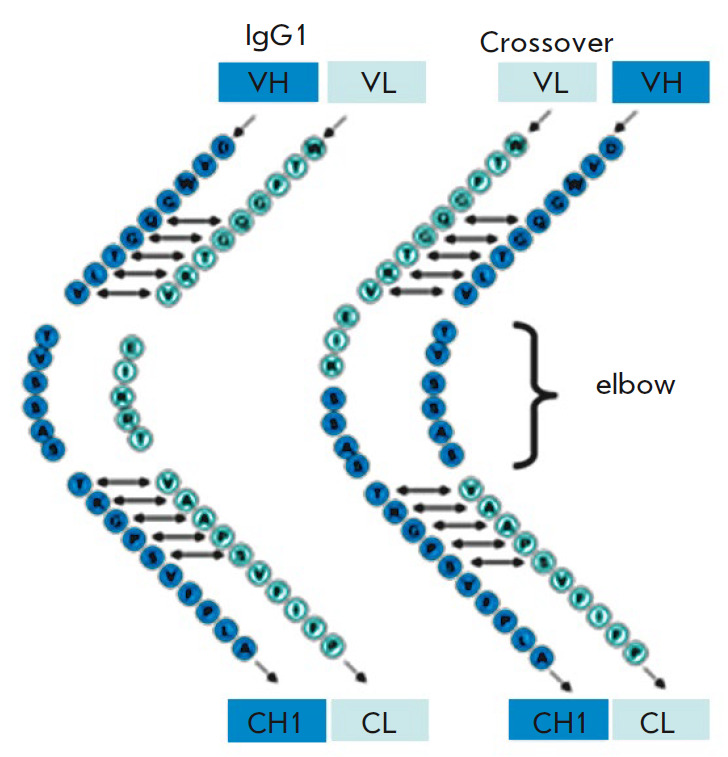
Schematic representation of the crossover of the amino acid sequences of the antibody H- and L-chain variable regions (elbow region) [29]

To prevent improper pairing of L- and H-chains of different antibodies, W. Schaefer et al. [30] proposed an elegant solution called the “crossover” of antibody variable domains. The antibody VL domain is attached to the H chain CH1 domain, and the VH domain is attached to the L chain kappa domain in the so-called elbow region (*Fig. 1*). The amino acid sequences in the crossover region are described in more detail in [30].

**Fig. 2:**
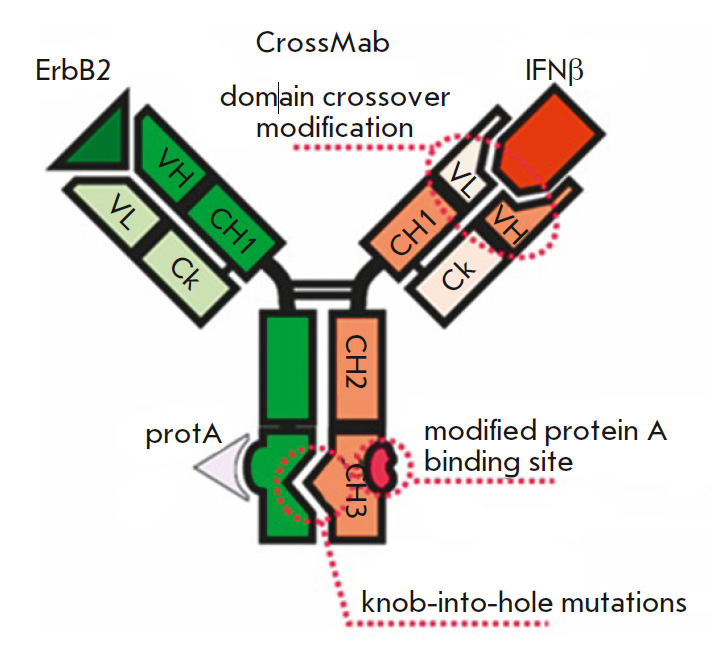
Structure of a bispecific antibody with crossover of the variable domains in the IFNβ binding region, knob-hole mutations, and mutations reducing binding to *Staphylococcus aureus *protein A

In our study, the crossover was planned for a BsAb region corresponding to the B16 IFNβ-neutralizing antibody. Hole-type mutations were introduced into the H chain corresponding to the anti-ErbB2 antibody; knob*-type mutations were located in the B16 antibody H chain. This enables preferential binding of H-chains as knob-hole pairs, thereby ensuring the formation of a bispecific antibody. Schematically, the structure of a bispecific antibody with a crossover of variable domains, knob*-hole mutations in the H chain CH_3_ domain, and mutations reducing binding to protein A-sepharose is shown in *Fig. 2*.

**Fig. 3:**
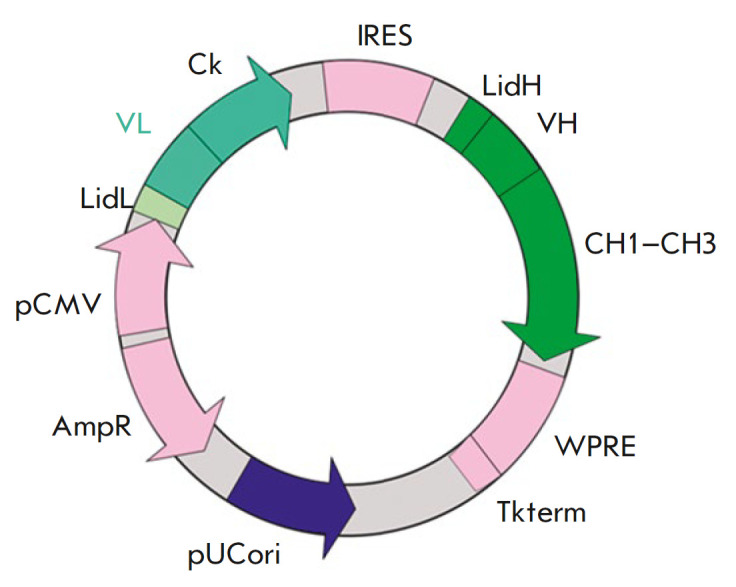
Schematic of a bicistronic vector for BsAb expression. pCMV – cytomegalovirus promoter; *VH *and *VL *– variable antibody domain genes; *CH1*–*CH_3_*– sequences encoding the constant domains of the antibody H-chain; IRES – internal ribosome entry site; WPRE – regulatory element sequence; Tkterm – signal sequence of polyadenylation of the herpes simplex virus thymidine kinase mRNA

To simultaneously produce four chains – L and H antibodies to ErbB2 as well as L and H antibodies to IFNβ –we used bicistronic vectors in which the genes of the heavy and light chains of each antibody were expressed under the control of a single promoter. The pcDNA3.4 Poly40 plasmid (a proprietary modification of the pcDNA3.4 vector (Invitrogen) with an inserted polylinker) was chosen as an expression vector for the production of BsAbs in eukaryotic cells. This plasmid has a cytomegalovirus immediate-early promoterenhancer to ensure biosynthesis of the target protein. Given the results of our previous studies, the promoter was first followed by the antibody L chain gene; then, the H chain gene was placed after the IRES regulatory element. An internal ribosome entry site (IRES) of the encephalomyocarditis virus (EMCV) was used as a regulatory element for ensuring expression of the antibody H chain gene (*Fig. 3*).

Two such vectors were prepared. In one vector, sequences encoding the L chain and H chain with holetype mutations in the trastuzumab antibody were cloned; in the other, sequences encoding the L chain and H chain with knob*-type mutations in the human anti- IFNβ-1a antibody were cloned in the crossover format. To facilitate the purification of the bispecific antibody by affinity chromatography, the H457R and Y458F mutations were introduced into the coding sequence of the CH_3_ domain of the H chain gene containing knob-type mutations. Combinations of two vectors were used for transient expression in mammalian cells, which produced bispecific antibodies to two different antigens.

**Fig. 4:**
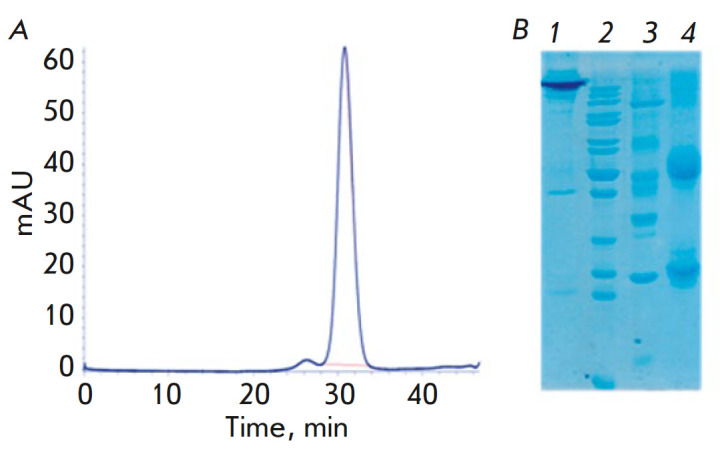
Chromatogram of the B16 BsAb in the CrossMab format (*A*). Electrophoregram of the B16 BsAb in 12% SDS-PAGE; (*B*): 1 – non-reducing conditions; 4 – reducing conditions; 2, 3 – molecular weight markers: 2 – 200.0, 150.0, 120.0, 100.0, 85.0, 70.0, 60.0, 50.0, 40.0, 30.0, 25.0, 14.4 kDa; 3 – 116.0, 66.2, 45.0, 35.0, 25.0, 14.4 kDa

Antibodies were produced using a transient expression system. A transient expression system in CHO cells enables fast production of recombinant antibodies in an amount sufficient for an immunochemical analysis, a determination of affinity, and investigation of the neutralizing activity. In this case, the proteins have the correct spatial folding and are correctly glycosylated. After purification on a protein-A-sepharose affinity column, analytical gel-filtration was performed on a Superdex 200-10/300 GL resin to detect the antibodies and determine their ratio of monomeric and oligomeric forms. Analytical HPLC demonstrated that the introduction of a second chromatographic step increases the purity of recombinant antibodies to 96–98% and enables purification from aggregate forms of the recombinant antibodies (*Fig. 4A*).

The purity and homogeneity of BsAbs was confirmed by Laemmli electrophoresis [31] under reducing and non-reducing conditions, followed by densitometry of the electrophoregram (*Fig. 4B*). According to the densitometry results, the purity of BsAbs was 97.8 ± 1.0%.

**Investigation of BsAbs by immunochemical methods**

**Fig. 5:**
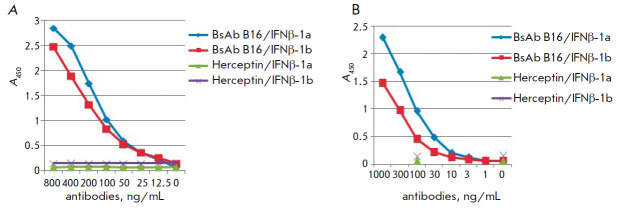
Binding of bispecific (*A*) and mouse monoclonal prototypical antibodies (*B*) to glycosylated (IFNβ-1a) and non-glycosylated (IFNβ-1b) IFNβ. Herceptin – commercial drug, antibody to the ErbB2 receptor (negative control)

*Binding of BsAbs to IFNβ. *Binding of BsAbs to the glycosylated and non-glycosylated forms of IFNβ was tested by indirect ELISA. Both the glycosylated (IFNβ-1a) and non-glycosylated (IFNβ-1b) forms of IFNβ adsorbed in a solid phase were found to bind to BsAbs (*Fig. 5*). The binding of BsAbs to IFNβ was similar to that of the mouse B16 antibody – a BsAb prototype.

**Fig. 6:**
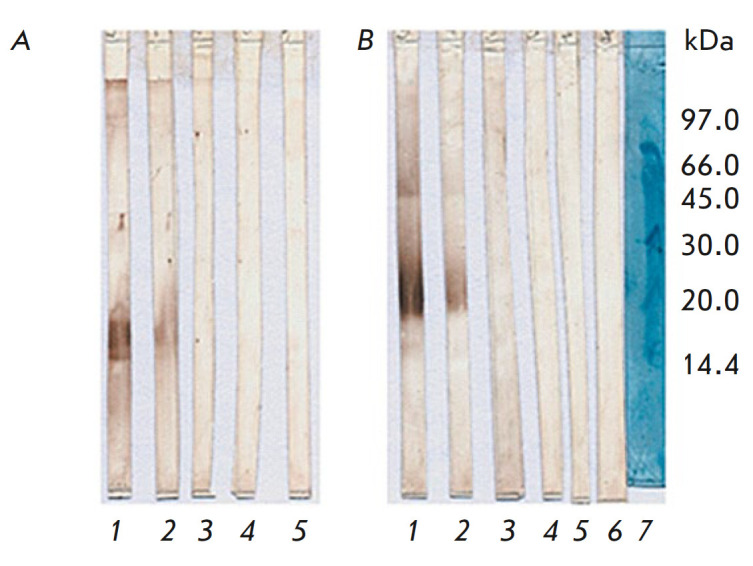
Immunoblot of IFNβ-1b (non-glycosylated; Betaferon) (*A*) and IFNβ-1a (glycosylated; Pharmapark) (*B*) with BsAb under non-reducing conditions after 15% SDS-PAGE. Lanes: 1 – BsAb B16/1; 2 – BsAb B16/2; 3 – Herceptin; 4 – negative control (humanized antibodies against Shiga toxin); 5 – control of an anti-species peroxidase conjugate of the 4g7 antibody against the human Ig kappa chain; 6 – trastuzumab; 7 – molecular weight markers

Binding of BsAbs to IFNβ was also examined by immunoblotting (Western blotting). This method enables visualization of antigen–antibody immune complexes after electrophoretic separation of the antigen sample under denaturing conditions, followed by transfer to the membrane. The main difference between immunoblotting and indirect ELISA is the ability to discriminate between immune complexes with different molecular weights, e.g., monomeric and oligomeric forms of antigen as well as intact antigen, and products of its degradation. At the first stage of immunoblotting, IFNβ samples were electrophoretically separated in 15% SDS-PAGE under non-reducing conditions. As previously shown, IFNβ under reducing conditions loses its ability to bind to antibodies (data not shown); therefore, immunoblotting under reducing conditions was not performed. Immunoblotting confirmed the specificity of the analyzed BsAbs, which was earlier demonstrated by indirect ELISA—antibody samples interacted with glycosylated IFNβ (Pharmapark) and non-glycosylated IFNβ (Betaferon) and stained bands with molecular weights of 18.5 kDa (non-glycosylated IFNβ) and 20–22 kDa (glycosylated IFNβ) (*Fig. 6*).

**Fig. 7:**
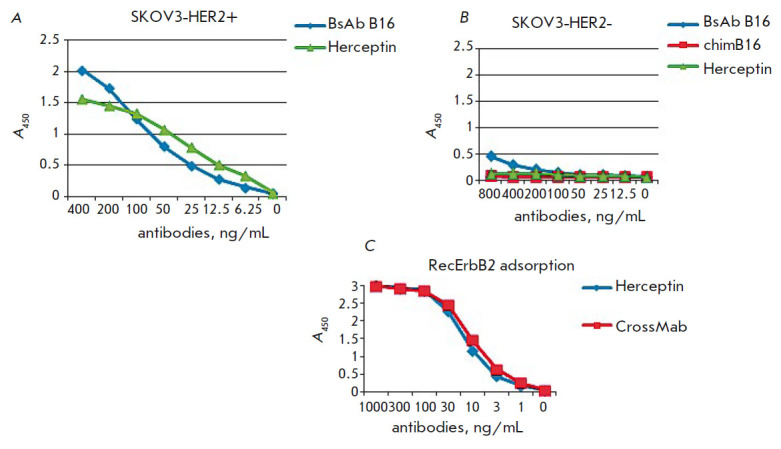
Titration curves of the BsAb B16 and the control antibody Herceptin on the lysates of the HER2-overexpressing cell line SKOV3 (*A*), HER2-underexpressing cell line SKOV3 (*B*), and the recombinant extracellular domain of the ErbB2 receptor (*C*)

*Binding of BsAbs to ErbB2. *The produced BsAbs should exhibit reactivity not only towards IFNβ, but also towards a surface molecule of tumor cells – ErbB2. Binding of BsAbs to ErbB2 was analyzed by indirect ELISA using cell lysates. The pharmaceutical drug Herceptin (trastuzumab) was used as a positive control. The ELISA was performed on lysates of tumor cell lines both expressing and non-expressing ErbB2 (*Fig. 7A,B*). The ErbB2 expression level was evaluated by indirect ELISA using cell lysates and Herceptin (data not shown). The inflection point (EC_50_) of the Herceptin antibody titration curve was 15 ng/mL for the ErbB2-expressing SKOV3 line and more than 3 000 ng/mL for the ErbB2-negative SKOV3 line.

Bispecific antibodies interacted with the cell lysates of the ErbB2-positive SKOV3 line (*Fig. 7A*) and did not interact with the lysates of SKOV3 cells with low HER2 expression (*Fig. 7B*). Also, binding of BsAbs to ErbB2 was shown by ELISA with solid phase adsorption of a recombinant ErbB2 extracellular domain obtained in our laboratory, compared to the commercial drug Herceptin specific to this receptor. Binding of the Cross- Mab BsAb to the receptor was the same as binding of the Herceptin antibody (*Fig. 7C*).

**Analysis of simultaneous binding of interferon-β and the ErbB2 receptor by sandwich ELISA**

**Fig. 8:**
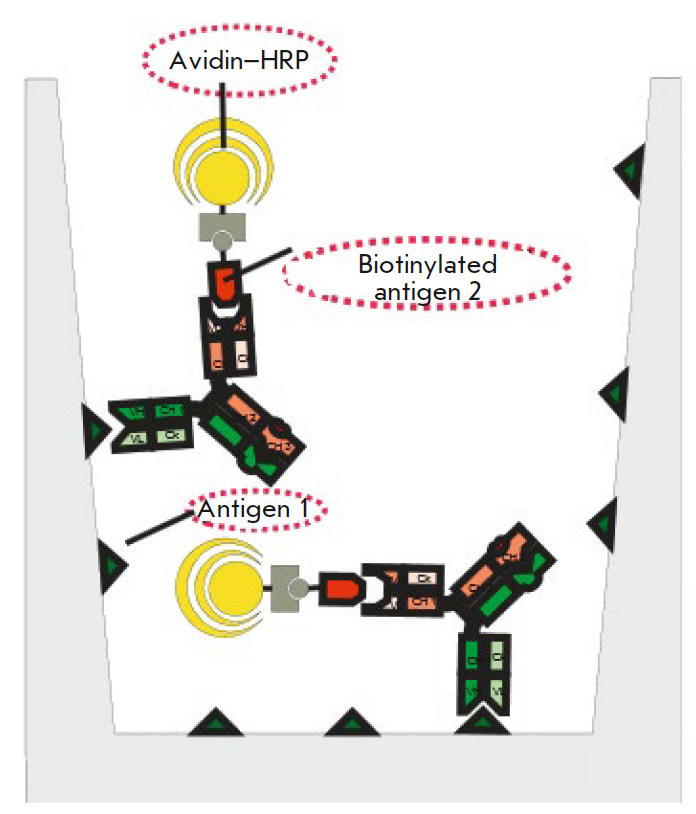
Scheme of a sandwich ELISA for the analysis of the simultaneous binding of BsAb to IFNβ and the ErbB2 receptor

**Fig. 9:**
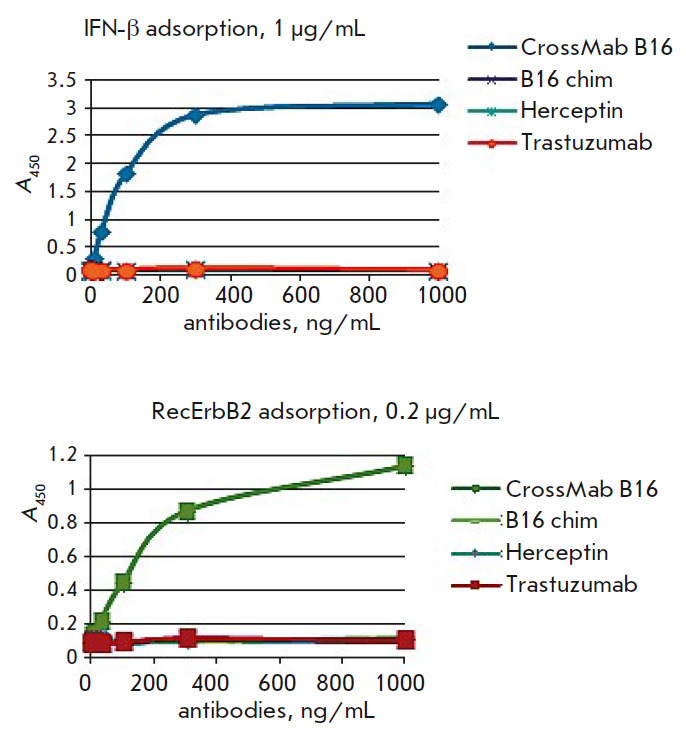
Titration curves of BsAb in sandwich ELISA compared to the prototype chimeric monospecific antibody B16 against IFNβ and monospecific antibodies against the ErbB2 receptor. CrossMab B16 – a full-length bispecific antibody against the ErbB2 receptor and IFNβ

Bispecific binding of the produced antibody was confirmed by sandwich ELISA in two variants (*Fig. 8*). In the first variant, IFNβ (antigen 1) was adsorbed onto a solid phase and then, after blocking, incubated with BsAbs in serial dilutions. After the washing of unbound odies, biotinylated recombinant ErbB2 (antigen 2) and an avidin–horseradish peroxidase conjugate were added. In the second variant, recombinant ErbB2 (antigen 1), BsAbs (in serial dilutions), biotinylated IFNβ (antigen 2), and an avidin–horseradish peroxidase conjugate were sequentially adsorbed onto a solid phase. Monospecific antibodies were used as a control: a B16 chimeric neutralizing antibody to IFNβ and antibodies to ErbB2 – the commercial drug Herceptin and the produced recombinant antibody trastuzumab (Herceptin analogue). In the case of the monospecific antibodies, no ELISA signal was observed. The bispecific antibody bound to IFNβ and ErbB2 in both variants (*Fig. 9*).

The bispecific nature of the produced BsAbs was confirmed by sandwich ELISA.

**Interferon-β-neutralizing activity of bispecific antibodies**

The biological activity of the BsAb samples was evaluated in experiments on the neutralization of the antiproliferative effect of IFNβ. The bispecific antibodies used as a means of IFNβ delivery to tumor cells should have neutralizing properties against the cytokine to avoid adverse systemic reactions during transport. Binding of the immunocytokine complex to tumor cells is supposed to lead to the release of IFNβ and the onset of its antiproliferative effect, in addition to the action of the anti-ErbB2 antibody. The antibody’s ability to neutralize IFNβ was evaluated using a cell model lacking ErbB2. Due to the antiproliferative activity of the BsAb region responsible for binding to ErbB2, it was not possible to analyze the antiproliferative activity of IFNβ by another method. The experiments were performed on the colon adenocarcinoma cell line HT29 that is the most sensitive, according to preliminary experiments, to the antiproliferative effect of IFNβ (results not shown). Recombinant glycosylated IFNβ was added to serial dilutions of BsAbs and the control antibodies: the mouse B16 IFNβ-neutralizing antibody, as well as chimeric and humanized B16-based antibodies, as a positive control. Tumor cells were cultured in a mixture with PBMCs in the presence of IFNβ and antibodies at various concentrations. Cultivation was carried out for 5 days. The number of living cells was evaluated by the MTT test [27]. The neutralizing activity of the antibodies was expressed as a percentage of the cell proliferation rate without IFNβ and calculated by the formula

neutralization (%) = (*A*_i_ – *A*_0_)/(*A*_100_ – *A*_0_) × 100%,

where *A*_i_ is the mean optical density in the wells with an *i*-th antibody concentration; *A*_0_ is the mean optical density in the wells with IFNβ without antibodies; *A*_100_ is the mean optical density in the wells without IFNβ and antibodies.

**Fig. 10:**
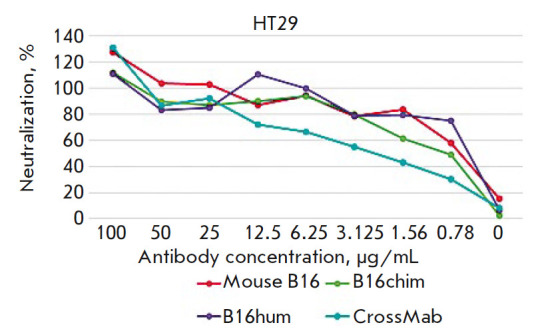
Neutralization of the antiproliferative effect of interferon-beta by bispecific antibodies in the CrossMab format compared to prototypical mouse B16 antibodies, chimeric (B16chim) antibodies, and humanized (B16hum) antibodies

On the basis of our analysis (*Fig. 10*), the neutralization index IC_50_ for the B16 BsAb was found to be 50 μg/mL, which is 2.5-fold higher than that for the prototype mouse B16 antibody. This may be explained by the fact that the BsAb contains only one interferonbinding site, while the mouse antibody contains two. In addition, the B16 BsAb lacks a cooperative binding effect, so that its neutralizing activity corresponds to the theoretically assumed one.

## CONCLUSION

We used the B16 neutralizing antibody to IFNβ and the trastuzumab (Tz) antibody specific to ErbB2 to produce bispecific CrossMab antibodies with knob and hole mutations in the CH_3_ domain of H chains. These proteins were shown to bind and neutralize IFNβ, as well as ErbB2, in tumor cell lysates and as a recombinant extracellular domain. These molecules may be used as a component of the immunocytokine complex for the delivery of IFNβ to the cells of ErbB2-associated tumors, which may block the side effects caused by IFNβ monotherapy. This approach to the treatment of ErbB2-associated tumors will be tested in animal models.

## Abbreviations

ErbB2: human epidermal growth factor receptor 2
IFNβ: human interferon-beta
Tz: trastuzumab
mAb: monoclonal antibody
BsAb: bispecific antibody
L and H: antibody light and heavy chains
VL and VH: antibody light and heavy chain variable domains
CH1–CH3: antibody heavy chain constant domains
SOE-PCR: PCR with overlapping regions
EC50: half maximal effective concentration
PBS: phosphate-saline buffer

## References

[1] https://nmicr.ru/meditsina/onkologicheskie-zabolevaniya-i-programmy-lecheniya-raka/programma-protiv-rakaverkhnikh-dykhatelnykh-putey-i-grudnoy-kletki/rakmolochnoy-zhelezy/.

[2] Ross J.S., Fletcher J.A., Linette G.P., Stec J., Clark E., Ayers M., Symmans W.F., Pusztai L., Bloom K.J. (2003). Oncologist..

[3] Owens M.A., Horten B.C., Da Silva M.M. (2004). Clin. Breast Cancer..

[4] Moasser M.M. (2007). Oncogene..

[5] Slamon D.J., Godolphin W., Jones L.A., Holt J.A., Wong S.G., Keith D.E., Levin W.J., Stuart S.G., Udove J., Ullrich A., Press M.F. (1989). Science..

[6] Scholl S., Beuzeboc P., Pouillart P. (2001). Ann. Oncol..

[7] Yan M., Schwaederle M., Arguello D., Millis Sh.Z., Gatalica Z., Kurzrock R. (2015). Cancer Metastasis Rev..

[8] Gerstein E.S., Kushlinsky N.E., Davydov M.I. (2010). Molecular medicine..

[9] Chantry A. (1995). J. Biol. Chem..

[10] Diermeier S., Horvath G., Knuechel-Clarke R., Hofstaedter F., Szollosi J., Brockhoff G. (2005). Exp. Cell Res..

[11] Vogel C.L., Cobleigh M.A., Gutheil J.C., Harris L.N., Fehrenbacher L., Slamon D.J., Murphy M., Novotny W.F., Burchmore M., Shak S. (2002). J. Clin. Oncol..

[12] Figueroa-Magalhães M.C., Jelovac D., Connolly R.M., Wolff A.C. (2014). Breast..

[13] Damdinsuren B., Nagano H., Sakon M., Kondo M., Yamamoto T., Umeshita K., Dono K., Nakamori S., Monden M. (2003). Ann. Surg. Oncol..

[14] Rosenblum M.G., Yung W.K., Kelleher P.J., Ruzicka F., Steck P.A., Borden E.C. (1990). J. Interferon Res..

[15] Vitale G., van Eijck C.H., van Koetsveld Ing. P.M., Erdmann J.I., Speel E.J., van der Wansem Ing. K., Mooij D.M., Colao A., Lombardi G., Croze E. (2007). Ann. Surg..

[16] Horikoshi T., Fukuzawa K., Hanada N., Ezoe K., Eguchi H., Hamaoka S. (1995). J. Dermatol..

[17] Wan S., Pestka S., Jubin R.G., Lyu Y.L., Tsai Y.C., Liu L.F. (2012). PLOS One..

[18] Borden E.C. (2019). Nat. Rev. Drug Discovery..

[19] Dubrot J., Palazón A., Alfaro C., Azpilikueta A., Ochoa M.C., Rouzaut A., Martinez-Forero I., Teijeira A., Berraondo P., Le Bon A. (2011). Int. J. Cancer..

[20] Trinh K.R., Vasuthasawat A., Steward K.K., Yamada R.E., Timmerman J.M., Morrison S.L. (2013). J. Immunother..

[21] Yang X., Zhang X., Fu M.L., Weichselbaum R.R., Gajewski T.F., Guo Y., Fu Y.X. (2014). Cancer Cell..

[22] Pogue S.L., Taura T., Bi M., Yun Y., Sho A., Mikesell G., Behrens C., Sokolovsky M., Hallak H., Rosenstock M.Yu. (2016). PLOS One..

[23] Li Z., Zhu Y., Li C., Trinh R., Ren X., Sun F., Wang Y., Shang P., Wang T., Wang M. (2017). Oncoimmunology..

[24] Aliev T.K., Dolgikh D.A., Kirpichnikov M.P., Panina A.A., Rybchenko V.S., Sveshnikov P.G., Solopova O.N., Toporova V.A., Shemchukova O.B. (2018). A monoclonal antibody capable of neutralizing the biological activity of human interferon beta-1A. Patent application No. 2018147193 of December 28, 2018..

[25] https://www.drugbank.ca.

[26] Panda S.K., Ravindran B. (2013). Bio-protocol..

[27] Mosmann T. (1983). J. Immunol. Methods..

[28] Atwell S., Ridgway B.B., Wells J.A., Carter P. (1997). J. Mol. Biol..

[29] Tustian A.D., Endicott C., Adams B., Mattila J., Bak H. (2016). MABS..

[30] Schaefer W., Regula J.T., Bähner M., Schanzer J., Croasdale R., Dürr H., Gassner C., Georges G., Kettenberger H., Imhof-Jung S. (2011). Proc. Natl. Acad. Sci. USA..

[31] Laemmli U.K. (1970). Nature.

